# Breathing New Life: Nursing Perspectives on Overcoming Lung Transplantation Challenges

**DOI:** 10.7759/cureus.83639

**Published:** 2025-05-07

**Authors:** Devanshi Chowdhary, Suman Dubas, Ritu Rani, Vanita Kumari

**Affiliations:** 1 Nursing, All India Institute of Medical Sciences, Rishikesh, Rishikesh, IND; 2 Nursing, All India Institute of Medical Sciences, New Delhi, New Delhi, IND

**Keywords:** broncheictasis, bronchiectasis, infection, lung, lung transplant, transplant, ventilation

## Abstract

Bronchiectasis is a complex airway disease marked by permanent dilation of the bronchial tubes, leading to chronic respiratory symptoms, repeated lung infections, progressive lung function decline, and ultimately, chronic illness and premature death. Lung transplantation is an established treatment for various end-stage lung diseases. However, the process of lung transplantation presents significant challenges, often due to the patient's already weakened health, frequent infections, and prolonged treatment requirements. This report presents the first documented case of lung transplantation at a major public sector hospital in northern India, in a patient with symptomatic cystic bronchiectasis and recurrent hospitalizations since the age of 12. We explore the range of nursing and medical challenges encountered during the patient's hospital stay and the measures taken to achieve a successful transplant despite these obstacles.

## Introduction

Bronchiectasis is a Greek word derived from “bronkos” meaning airway and “ectasis” meaning widening [1]. It is a chronic lung condition, defined as the abnormal, irreversible dilatation of the bronchi where the elastic and muscular tissue is destroyed by acute or chronic inflammation and infection [2]. Unless appropriately managed, the combination of infection and chronic inflammation results in progressive lung damage [3]. It can be classified as cylindrical, follicular, cystic, or saccular bronchiectasis, which is also the most severe form of the disease. The bronchi dilate, forming large cysts, which are usually filled with air and fluid. Clinically, the disease manifests as recurrent episodes of respiratory tract infections together with an enduring cough and continuous production of sputum. The mainstay of treatment consists of non-pharmacological measures, e.g., bronchopulmonary hygiene and physical therapy and pharmacological therapy, such as antibiotics, anti-inflammatory therapy, and mucous mobilizing agents [4]. Lung transplant is the ultimate treatment for end-stage lung diseases and can be successfully performed on patients with advanced bronchiectatic lung disease, with subsequent good post-transplant quality of life and long-term outcome [5]. Various complications like graft dysfunction, acute rejection, postoperative infections, postoperative hemorrhage, leaks, large airway complications, and vascular anastomotic complications can occur post-transplant [6]. Intensive postoperative surveillance and holistic patient care are key to the successful management of lung transplantation patients with respect to early detection, treatment of infections, and successful transplantation [7]. The following case is the first documented case of lung transplant in one of the largest public sector hospitals in India; thus, it provides an important perspective on the care of patients undergoing lung transplantation.

## Case presentation

Pre-operative course of illness

The patient had been symptomatic since 12 years of age with frequent exacerbations, but her condition had worsened in the last four years with progressive shortness of breath (New York Heart Association (NYHA) III) and nasal continuous positive airway pressure (CPAP) support for the last 1½ years. Presently, the patient has been admitted with complaints of intermittent fever and a mucopurulent cough for the last two weeks. On physical examination, the patient was tachycardic, tachypneic, dyspneic (Medical Research Council (MRC) grade IV), with decreased air entry and bilateral lower lobe crepitations. Grade III clubbing, bilateral pedal edema, burning micturition, and decreased urinary output were also noted. Sputum cultures were positive for *Aspergillus terreus* infection. The patient was being treated with tablet voriconazole 200 mg twice daily (BD), mucolytic agents, an alternate regimen of oxygen therapy at 3 L/minute, and non-invasive ventilation (NIV) support (bilevel positive airway pressure (BiPAP) at 10/4 pressure settings). Despite the treatment, the patient had already slipped into hypercapnic respiratory failure.

Preoperative lung transplant evaluation

**Figure 1:**
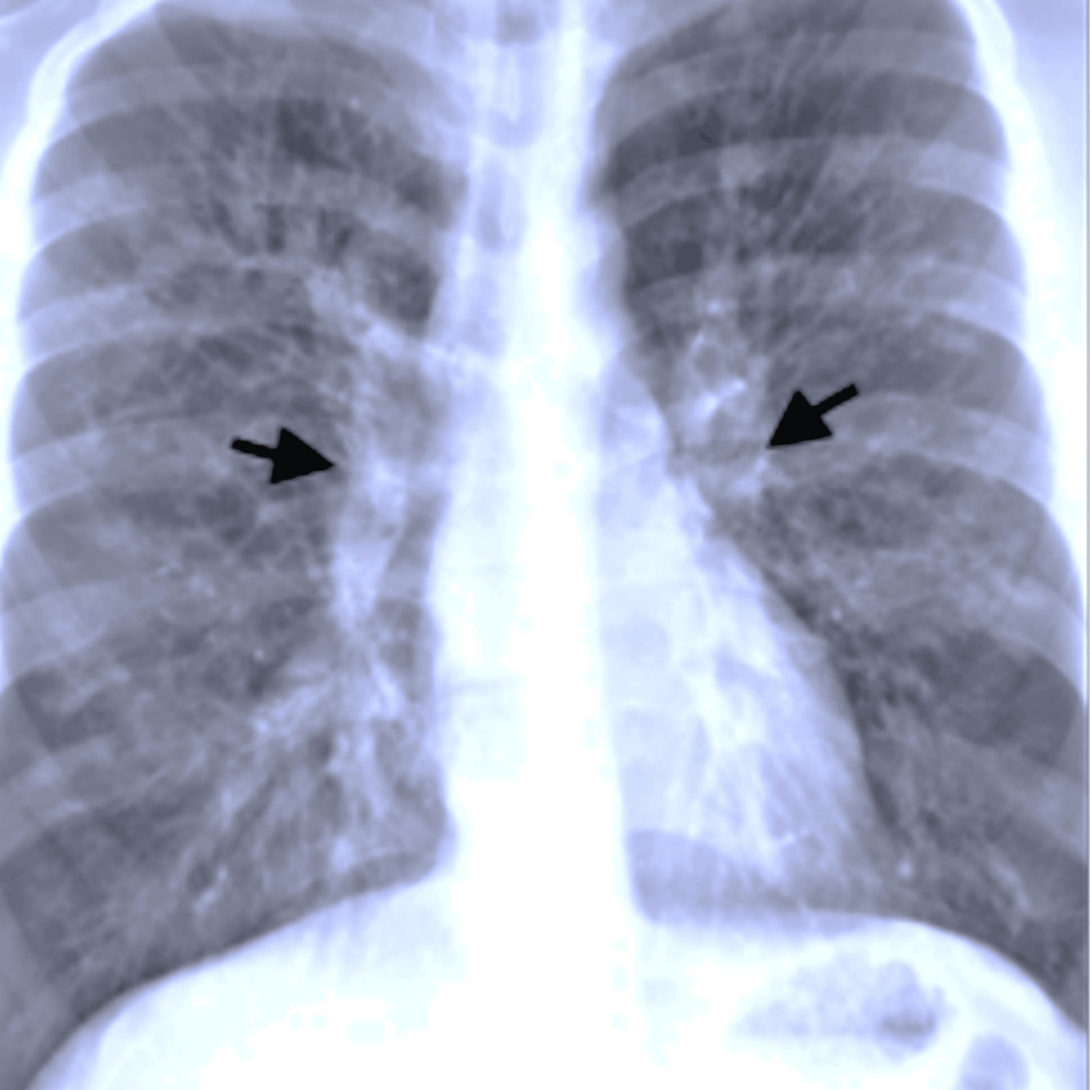
Preoperative chest X-ray revealing pulmonary plethora in the patient's native lung

While the patient was being treated, the patient was identified as a suitable suitor for a lung transplant from a 45-year-old male auto driver. An immediate preoperative assessment was done for the patient. An echocardiogram revealed right ventricular (RV) dysfunction, right atrial (RA) and RV dilation with moderate to severe pulmonary artery hypertension with a tricuspid annular plane systolic excursion (TAPSE) < 18 and ejection fraction of 60%. Right heart catheterization also revealed similar findings. The patient was MRC grade IV and was unable to complete the pulmonary function test (PFT); chest CT showed bilateral bronchial dilation with a broncho-arterial ratio greater than 1.5. A chest X-ray revealed pulmonary plethora (Figure 1).

Renal and hematological assessments were done, and they were within the normal range. A preoperative psychiatry consultation concluded that the patient was in good mental health, with a Mini-Mental State score of 15.

Preoperative preparation

Preoperatively, limb and lung physiotherapy were given the utmost importance. The transplant room was prepared to receive the transplant patient. It was deep cleaned and carbolized with 1% carbolic acid, fumigated with echoshield solution (hydrogen peroxide 11% w/v with 0.01% w/v diluted silver nitrate) thrice before the patient was transferred into the ICU.

The hospital regimen consisted of a cephalosporin glycopeptide antibiotic and immunosuppressant drugs. Tacrolimus (TAC) 0.5 mg and mycophenolate mofetil 500 mg were given preoperatively before taking the patient into the operating theater. Intraoperatively, doses of antibiotics were repeated at half-life. An injection of methyprednisolone 500 mg was given just before implantation of the donor lung.

Postoperative course

On 6/5/2022, the patient underwent off-pump bilateral orthotopic lung transplant and was received in the ICU sedated and paralyzed, on pressure-regulated volume control ventilation with FiO_2_ 100%, respiratory rate (RR) 14 breaths/minute, positive end-expiratory pressure (PEEP) 7 mmHg (range: 5-7 mmHg), tidal volume 300 mL, pressure support 8 mmHg. The patient was also on low-dose inotropic support with norepinephrine 0.1 µg/kg/minute. The sternum was kept open (in case of any emergency situation), with two mediastinal and two pleural drains in situ.

Inotropic support was tapered off the next day, and a target of maintaining central venous pressure (CVP) at 8-10 mmHg, systolic blood pressure (SBP) of 110-120 mmHg, and urinary output of 50 mL/hour or above was focused. On the second postoperative day, the sternum was closed as the patient's condition stabilized. The patient underwent an elective tracheostomy on the third postoperative day, anticipating difficult weaning due to signs of excessive exertion and hyperventilation, which were leading to respiratory alkalosis. Ventilation settings were aimed at maintaining the lowest FiO_2_ possible to maintain a partial pressure of oxygen of ≤80 mmHg and saturation of 90-95%. Throughout the patient's stay, a low tidal volume of 6 mL/kg, low PEEP (5-7 cm H_2_O), and a pressure support of no more than 10 cm H_2_O were maintained, with an allowable plateau pressure kept below 25 cm H_2_O. Gradual weaning was implemented for the patient.

On the 10th postoperative day, the patient developed subcutaneous emphysema, leading to respiratory distress, which required reinitiation of pressure-regulated volume control (PRVC) mode. The chest tube was repositioned, and a small blow-hole incision was made in the infraclavicular area. Over the following month, the patient was gradually weaned to room air on tracheostomy, with improved lung compliance through regular lung and limb physiotherapy. The patient was then transferred to the step-down ward over the course of one month.

Nursing challenges during ICU hospitalization

Infection Prevention

A separate positive pressure room was designated for the patient, and infection control bundles were scrupulously followed. Air cultures were done bi-monthly, and barrier precautions were followed by all the healthcare workers working with the patient. A doctor and two nurses were assigned specifically for the patient in every shift, where one nurse was assigned as a circulator nurse. No relatives of the patient were allowed during the acute phase of the patient’s stay in the ICU (first seven postoperative days). Separate new dusters and mops were used for daily carbolization (carbolic acid at a concentration of 1:10) and mopping (5% carbolic acid) of the room, respectively. No healthcare worker with an upper respiratory tract infection was allowed inside the unit.

Other practices followed were regular handwashing, using boiled utensils and boiled water for the patient, and oral hygiene by regular chlorhexidine mouthwash (0.2%) after having food items or otherwise every six hours.

Tracheal hygiene was upheld through regular, as-needed suctioning with minimal suction pressure (below 80 mmHg), performed either by a pulmonologist or by a nurse/physiotherapist under the supervision of a pulmonologist. Blood and urine tests (culture sensitivity and routine) were sent on every second day to check the infection as soon as possible. Despite considerable infection precautions and an extensive antibiotic regimen, the patient developed hospital-acquired pneumonia (*Klebsiella pneumoniae*) due to the prolonged weaning process. Polymyxin B, 5 lakh IU, was started thereafter intravenously BD.

Decreased Respiratory Muscle Strength

Due to the prolonged disease process and CPAP support preoperatively, which had led to decreased respiratory muscle strength, weaning of the patient was a big challenge. Lung physiotherapy emphasizing pulmonary toileting was done regularly, wherein breathing exercises like apical segmental and intercostal breathing, huffing, percussion, postural drainage, and ambulation were mostly emphasized. In the later phase of the ICU stay, active exercises like walking and pedal bicycling were also done twice a day. To relieve muscle tension and maintain muscle strength, the patient was kept on synchronized intermittent mandatory ventilation (SIMV) mode of ventilation during the night.

Decreased Weight and Dietary Intake

Due to the decrease in weight and the decreased intake of food, the patient was started on tube feeding with Fresubin 150 mL every three hours, accounting for 2000 kcal and 100 g of protein. The patient was later switched to oral feeds with a daily intake of a minimum of 1800 kcal and 80 g of protein. The food intake was duly noted by the nursing staff and was calculated every day by the dietician to have the needed macronutrients.

Postoperative Evaluation

**Figure 2:**
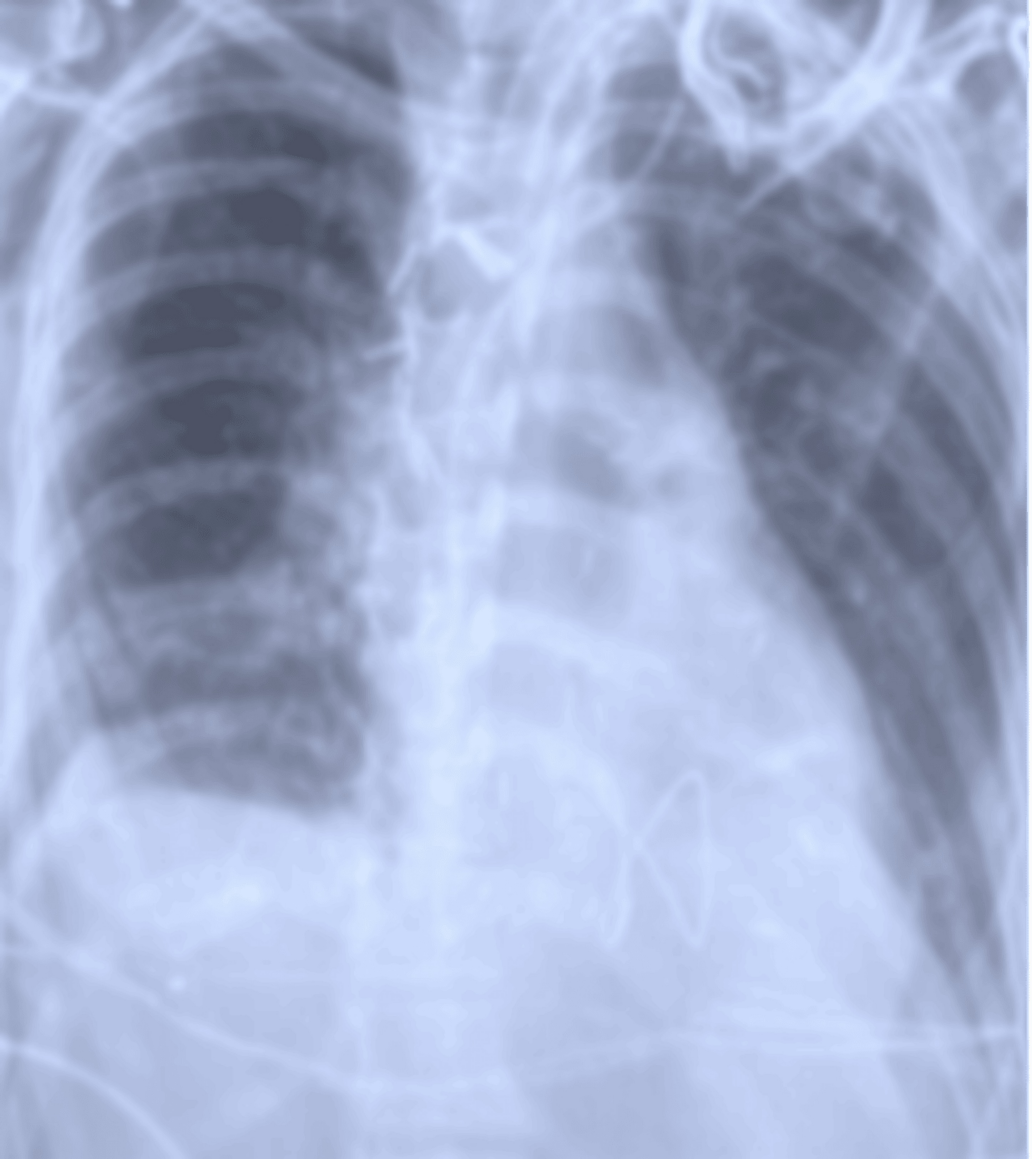
Postoperative chest X-ray, showing considerably clear lung fields

The patient was managed conservatively with regular hemograms, renal function, liver function, and a coagulation profile assessed daily. TAC level was checked twice a week, and the dose of TAC was titrated accordingly. The mean TAC level was maintained at 15-20 ng/mL during the first two postoperative weeks, and at 10-15 ng/mL thereafter, ensuring that the TAC level remained consistently above 10 ng/mL on all days. Postoperatively, chest X-ray was done every alternate day or as needed during the patient's stay in the ICU (Figure 2).

Discharge Planning

On 10/7/22, the patient's tracheostomy was decannulated, after which focus was further increased on chest physiotherapy. Once the patient was moved to a step-down ward, the patient was allowed self-administration of medications under the supervision of the nurse. The patient was informed about the warning signs: increasing shortness of breath, high-grade fever, recurrent cough, congestion, constant weight loss, and was instructed to report in case of any of these symptoms post-discharge. A handbook with the normal TAC level, WBC count, medications, doses, side effects, precautions (food, water, animal, respiratory, sexual practices, travel safety to be taken in public places, and use of mask) was handed over to the patient, which also consisted of columns where regular TAC level and hemogram values could be entered. A monthly follow-up was done for the next six months. Telephonic follow-up was done weekly. Second weekly TAC and hemogram levels were instructed to be done by the patient in any nearby hospital facility post-discharge for the first six months and every month thereafter.

Cystic bronchiectasis unresponsive to empirical treatment, bronchopulmonary hygiene, physical therapy, and pharmacological therapy like antibiotics, anti-inflammatory therapy, and mucous mobilizing agents is one of the indications of lung transplantation [5]. The patient presented with peculiar signs of cystic bronchiectasis, including high-grade fever, a productive cough (mucoid and relatively odorless), dyspnea (MRC IV) [8], and *Aspergillus terreus *infection, which is a common cause of exacerbation in these patients [9].

## Discussion

A preoperative evaluation of blood match, lung size match, right heart catheterization, chest CT, and cardiac evaluation is the mainstay of evaluation [10,11] and was done for the patient. Although primary graft dysfunction, which is one of the major complications with an incidence rate of 57%, was not seen in the patient [12]. Subcutaneous leak and infections [6] were encountered, which also became one of the major reasons for prolonged weaning and subsequent hospitalization of the patient. Respiratory muscle weakness is a common long-term complication in patients with bronchiectasis [13] and was seen in our patient, resulting in difficult weaning. Important consideration is given to reduce post-transplant infections by scrupulously following bundles for healthcare-associated infections [14]. The patient was kept under strict infection control monitoring to reduce the chances of any infection. It is a documented fact that the TAC level in a lung transplant patient should be kept above the level of 10 ng/mL in the first month of transplant [15]. In our patient, too, it was kept certain that the TAC level was always above 15 mg/dL. Lung protective ventilation is a documented regimen for lung transplant patients. This includes a low tidal volume (6-7 mL/kg), low PEEP (6-8 cm of H_2_O), and a maximum acceptable P plateau of 30 mmHg. Similar ventilatory settings were used in the patient, which have been shown to decrease pulmonary complications in these patients [12]. Lung and limb physiotherapy helps in the quick recovery of the patient in lung transplant [16], and thus, these measures were followed without exception. Dietary requirements of the patient were consciously managed; there is evidence that a transplant patient needs 50 to 60% more calories and twice as much protein in their diets than healthy individuals of the same age [17]. A holistic care with a multidisciplinary approach to the patient led to the successful lung transplant and subsequent discharge of the first lung transplant patient in a public sector hospital.

## Conclusions

Lung transplantation remains the mainstay of treatment in end-stage lung diseases, which are unresponsive to empirical treatment. The major concerns faced during the patient's stay were prolonged weaning and frequent infections. A multidisciplinary, holistic approach, with a strong willpower of the patient, was the most important pillar that led to the successful discharge of the patient. A detailed discharge teaching and easy accessibility of the patient to the hospital were maintained for the patient post-discharge.
